# 5-Methyl-2-pyridone

**DOI:** 10.1107/S1600536811033484

**Published:** 2011-08-27

**Authors:** Shulin Mao, Luo Yanghui, Pan Meiling

**Affiliations:** a Ordered Matter Science Research Center, College of Chemistry and Chemical Engineering, Southeast University, Nanjing 210096, People’s Republic of China

## Abstract

The crystal structure of the title compound, C_6_H_7_NO, is stabilized by inter­molecular N—H⋯O hydrogen bonds, resulting in inversion dimers. The structure is further consolidated by weak C—H⋯O hydrogen bonds.

## Related literature

For related structures, see: Boris-Marko *et al.* (2008 ▶); Vovk *et al.* (2003 ▶).
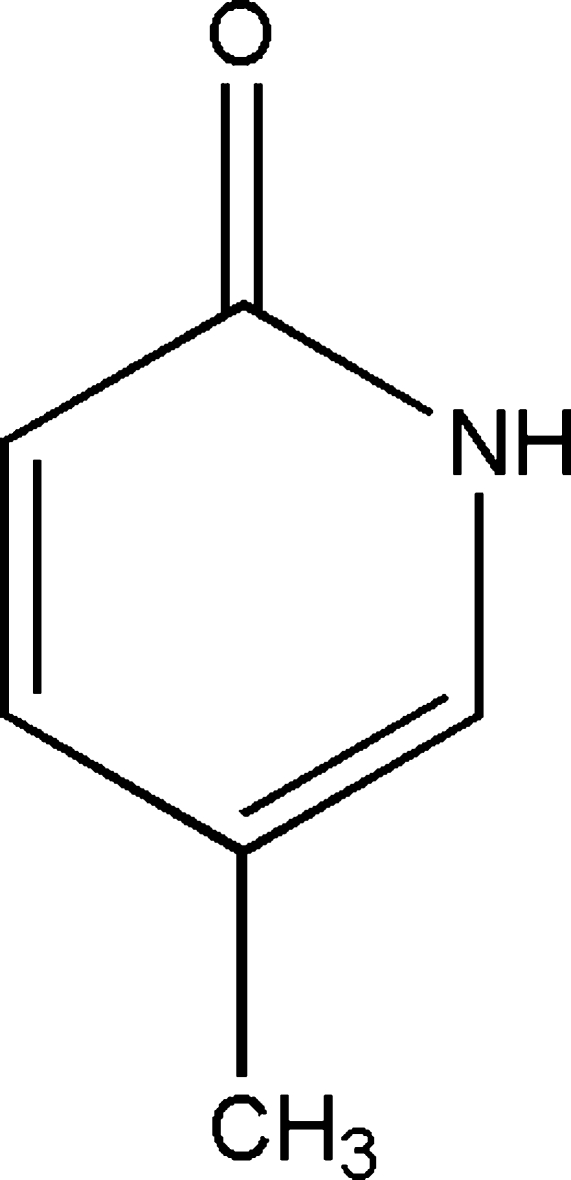


## Experimental

### 

#### Crystal data

C_6_H_7_NO
*M* *_r_* = 109.13
Monoclinic, 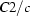

*a* = 12.965 (3) Å
*b* = 9.7154 (19) Å
*c* = 10.908 (2) Å
β = 118.96 (3)°
*V* = 1202.3 (4) Å^3^
*Z* = 8
Mo *K*α radiation
μ = 0.08 mm^−1^
*T* = 293 K
0.30 × 0.23 × 0.20 mm

#### Data collection

Rigaku SCXmini diffractometer
Absorption correction: multi-scan (*CrystalClear*; Rigaku, 2005 ▶) *T*_min_ = 0.977, *T*_max_ = 0.984
5961 measured reflections
1369 independent reflections
670 reflections with *I* > 2σ(*I*)
*R*_int_ = 0.049

#### Refinement

*R*[*F* ^2^ > 2σ(*F* ^2^)] = 0.054
*wR*(*F* ^2^) = 0.163
*S* = 0.99
1369 reflections
73 parameters
H-atom parameters constrained
Δρ_max_ = 0.12 e Å^−3^
Δρ_min_ = −0.17 e Å^−3^

### 

Data collection: *CrystalClear* (Rigaku, 2005 ▶); cell refinement: *CrystalClear*; data reduction: *CrystalClear*; program(s) used to solve structure: *SHELXS97* (Sheldrick, 2008 ▶); program(s) used to refine structure: *SHELXL97* (Sheldrick, 2008 ▶); molecular graphics: *DIAMOND* (Brandenburg & Putz, 2005 ▶); software used to prepare material for publication: *SHELXL97*.

## Supplementary Material

Crystal structure: contains datablock(s) I, global. DOI: 10.1107/S1600536811033484/pv2442sup1.cif

Structure factors: contains datablock(s) I. DOI: 10.1107/S1600536811033484/pv2442Isup2.hkl

Supplementary material file. DOI: 10.1107/S1600536811033484/pv2442Isup3.cml

Additional supplementary materials:  crystallographic information; 3D view; checkCIF report

## Figures and Tables

**Table 1:** Hydrogen-bond geometry (Å, °)

*D*—H⋯*A*	*D*—H	H⋯*A*	*D*⋯*A*	*D*—H⋯*A*
N1—H1*A*⋯O1^i^	0.86	1.94	2.800 (2)	173
C3—H3*A*⋯O1^ii^	0.93	2.46	3.334 (3)	157
C5—H5*A*⋯O1^iii^	0.93	2.33	3.260 (3)	178

Symmetry codes: (i) 
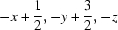
; (ii) 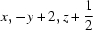; (iii) 
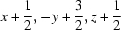
.

**Fig. 1.**
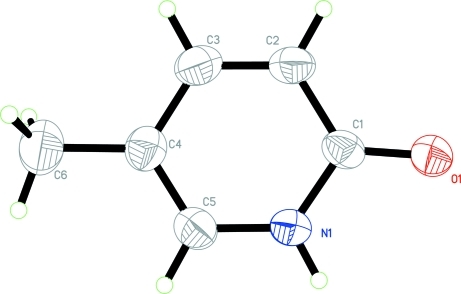
An ORTEP view of the title compound with the atom-numbering scheme. Displacement ellipsoids are drawn at the 30% probability level.

**Fig. 2.**
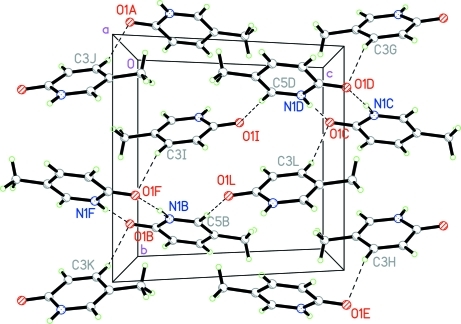
Unit cell packing of the title compound showing H-bonding interactions.

## supplementary crystallographic information

### Comment

The title compound is characterized by an enol-keto tautomerism due to the labile hydrogen atom of OH-group in α-position to the basic pyridine N atom which can easily migrate to N atom (Boris-Marko *et al.*, 2008) resulting in a zwitterionic molecule (Fig. 1).

The O1 and C6 atoms located on the pyridine ring are conplanar with the ring, deviating by 0.0.15 (3) and 0.35 (4) Å, respectively, from the ring plane, The crystal structure is stabilized by intermolecular N—H···O hydrogen bonds and further consolidated by C—H···O interactions (Fig.e 2 and Tab. 1).

### Experimental

To a solution of the title compounde (0.2 g) in acetone (2 ml) and ethanol (10 ml) was added was prepared by stirred at room temperature and then placed in a dark place. Colourless single crystals suitable for X-ray diffraction study were obtained by slow evaporation of the solution over a period of 8 d.

### Refinement

Positional parameters of all H atoms were calculated geometrically and refined using a riding model, with N–H = 0.086 Å and C—H = 0.93 and 0,96 Å for aryl and methyl type H-atoms, respectively, and *U*_iso_(H) = 1.2 *U*_eq_ (N/C-aryl) or 1.5 *U*_eq_ (C-methyl).

### Crystal data

**Table:**

C_6_H_7_NO	*F*(000) = 464
*M**_r_* = 109.13	*D*_x_ = 1.206 Mg m^−^^3^
Monoclinic, *C*2/*c*	Mo *K*α radiation, λ = 0.71073 Å
Hall symbol: -C 2yc	Cell parameters from 1369 reflections
*a* = 12.965 (3) Å	θ = 3.6–27.5°
*b* = 9.7154 (19) Å	µ = 0.08 mm^−^^1^
*c* = 10.908 (2) Å	*T* = 293 K
β = 118.96 (3)°	Prism, colourless
*V* = 1202.3 (4) Å^3^	0.30 × 0.23 × 0.20 mm
*Z* = 8	

### Data collection

**Table:**

Rigaku SCXmini diffractometer	1369 independent reflections
Radiation source: fine-focus sealed tube	670 reflections with *I* > 2σ(*I*)
graphite	*R*_int_ = 0.049
Detector resolution: 13.6612 pixels mm^-1^	θ_max_ = 27.5°, θ_min_ = 3.6°
CCD_Profile_fitting scans	*h* = −16→16
Absorption correction: multi-scan (*CrystalClear*; Rigaku, 2005)	*k* = −12→12
*T*_min_ = 0.977, *T*_max_ = 0.984	*l* = −14→14
5961 measured reflections	

### Refinement

**Table:**

Refinement on *F*^2^	Primary atom site location: structure-invariant direct methods
Least-squares matrix: full	Secondary atom site location: difference Fourier map
*R*[*F*^2^ > 2σ(*F*^2^)] = 0.054	Hydrogen site location: inferred from neighbouring sites
*wR*(*F*^2^) = 0.163	H-atom parameters constrained
*S* = 0.99	*w* = 1/[σ^2^(*F*_o_^2^) + (0.0734*P*)^2^] where *P* = (*F*_o_^2^ + 2*F*_c_^2^)/3
1369 reflections	(Δ/σ)_max_ < 0.001
73 parameters	Δρ_max_ = 0.12 e Å^−^^3^
0 restraints	Δρ_min_ = −0.17 e Å^−^^3^

### Special details

**Table:**

Geometry. All e.s.d.'s (except the e.s.d. in the dihedral angle between two l.s. planes) are estimated using the full covariance matrix. The cell e.s.d.'s are taken into account individually in the estimation of e.s.d.'s in distances, angles and torsion angles; correlations between e.s.d.'s in cell parameters are only used when they are defined by crystal symmetry. An approximate (isotropic) treatment of cell e.s.d.'s is used for estimating e.s.d.'s involving l.s. planes.
Refinement. Refinement of *F*^2^ against ALL reflections. The weighted *R*-factor *wR* and goodness of fit *S* are based on *F*^2^, conventional *R*-factors *R* are based on *F*, with *F* set to zero for negative *F*^2^. The threshold expression of *F*^2^ > σ(*F*^2^) is used only for calculating *R*-factors(gt) *etc*. and is not relevant to the choice of reflections for refinement. *R*-factors based on *F*^2^ are statistically about twice as large as those based on *F*, and *R*- factors based on ALL data will be even larger.

### Fractional atomic coordinates and isotropic or equivalent isotropic displacement parameters (Å^2^)

**Table:**

	*x*	*y*	*z*	*U*_iso_*/*U*_eq_	
N1	0.28530 (13)	0.79540 (16)	0.17990 (16)	0.0616 (5)	
H1A	0.3148	0.7521	0.1353	0.074*	
O1	0.11700 (11)	0.82537 (15)	−0.02634 (14)	0.0730 (5)	
C1	0.17371 (17)	0.8437 (2)	0.1049 (2)	0.0600 (6)	
C2	0.13100 (19)	0.9133 (2)	0.1852 (2)	0.0718 (7)	
H2A	0.0549	0.9488	0.1410	0.086*	
C5	0.35432 (18)	0.8107 (2)	0.3216 (2)	0.0672 (6)	
H5A	0.4300	0.7740	0.3655	0.081*	
C4	0.3147 (2)	0.8781 (2)	0.3988 (2)	0.0665 (6)	
C3	0.1989 (2)	0.9291 (2)	0.3250 (2)	0.0750 (7)	
H3A	0.1679	0.9755	0.3743	0.090*	
C6	0.3897 (2)	0.8989 (2)	0.5532 (3)	0.0978 (9)	
H6A	0.4652	0.8566	0.5840	0.147*	
H6B	0.3520	0.8578	0.6013	0.147*	
H6C	0.4000	0.9956	0.5734	0.147*	

### Atomic displacement parameters (Å^2^)

**Table:**

	*U*^11^	*U*^22^	*U*^33^	*U*^12^	*U*^13^	*U*^23^
N1	0.0493 (10)	0.0718 (12)	0.0626 (11)	0.0060 (8)	0.0263 (9)	−0.0035 (8)
O1	0.0544 (9)	0.1007 (12)	0.0615 (11)	0.0047 (7)	0.0262 (8)	0.0016 (8)
C1	0.0469 (12)	0.0662 (13)	0.0683 (15)	0.0002 (9)	0.0290 (12)	0.0079 (11)
C2	0.0635 (13)	0.0820 (16)	0.0773 (17)	0.0145 (11)	0.0401 (14)	0.0034 (12)
C5	0.0574 (13)	0.0666 (14)	0.0704 (15)	−0.0003 (10)	0.0252 (12)	−0.0006 (11)
C4	0.0713 (15)	0.0644 (14)	0.0624 (15)	−0.0024 (11)	0.0312 (13)	−0.0048 (11)
C3	0.0834 (17)	0.0739 (15)	0.0807 (18)	0.0098 (12)	0.0500 (15)	−0.0020 (12)
C6	0.109 (2)	0.102 (2)	0.0722 (18)	−0.0037 (14)	0.0354 (17)	−0.0110 (13)

### Geometric parameters (Å, °)

**Table:**

N1—C1	1.355 (2)	C5—H5A	0.9300
N1—C5	1.368 (2)	C4—C3	1.406 (3)
N1—H1A	0.8600	C4—C6	1.496 (3)
O1—C1	1.266 (2)	C3—H3A	0.9300
C1—C2	1.414 (3)	C6—H6A	0.9600
C2—C3	1.351 (3)	C6—H6B	0.9600
C2—H2A	0.9300	C6—H6C	0.9600
C5—C4	1.350 (3)		
			
C1—N1—C5	124.56 (18)	C5—C4—C3	115.9 (2)
C1—N1—H1A	117.7	C5—C4—C6	122.1 (2)
C5—N1—H1A	117.7	C3—C4—C6	122.0 (2)
O1—C1—N1	119.97 (19)	C2—C3—C4	122.6 (2)
O1—C1—C2	125.48 (19)	C2—C3—H3A	118.7
N1—C1—C2	114.55 (19)	C4—C3—H3A	118.7
C3—C2—C1	121.1 (2)	C4—C6—H6A	109.5
C3—C2—H2A	119.4	C4—C6—H6B	109.5
C1—C2—H2A	119.4	H6A—C6—H6B	109.5
C4—C5—N1	121.30 (19)	C4—C6—H6C	109.5
C4—C5—H5A	119.3	H6A—C6—H6C	109.5
N1—C5—H5A	119.3	H6B—C6—H6C	109.5

### Hydrogen-bond geometry (Å, °)

**Table:**

*D*—H···*A*	*D*—H	H···*A*	*D*···*A*	*D*—H···*A*
N1—H1A···O1^i^	0.86	1.94	2.800 (2)	173
C3—H3A···O1^ii^	0.93	2.46	3.334 (3)	157
C5—H5A···O1^iii^	0.93	2.33	3.260 (3)	178

Symmetry codes: (i) −*x*+1/2, −*y*+3/2, −*z*; (ii) *x*, −*y*+2, *z*+1/2; (iii) *x*+1/2, −*y*+3/2, *z*+1/2.
